# Kreosote in Erysipelas

**Published:** 1849-01

**Authors:** P. Fahnestock

**Affiliations:** Pittsburg


ARTICLE IX.—*Kreosote in Erysipelas.* By P. FAHNESTOCK, M. D., of Pittsburg. (Extracted from a letter to the Editor.)

Allow me to state that, during a practice of many years, I have been in the habit of using kreosote in erysipelas of the face, (as well as on all other parts of the body) in both its simple and phlegmonous forms, confining my local treatment to this article alone. And such has been the success of this treatment, that I have as yet to witness a case which has not yielded to it.

In every case of local erysipelas, I immediately apply the purest kreosote with a camel's hair brush over the whole of the affected surface, extending it some distance beyond the inflamed part, and at the same time administering a dose of chlor. hydrarg., followed by a sufficient portion of jalap to insure free catharsis. This, in the majority of cases, is all I find necessary. But when the mucuous membrane of the mouth and fauces is also affected, I pencil those parts with a strong solution of the nit. argent., say from ʒ ss. to ʒ i. to ℥ i. of distilled water.

In the phlegmonous form, it will be found necessary to repeat the application more frequently than in the simple, with the addition of a bread and water poultice, applied nearly cold and well sprinkled with water strongly impregnated with the kreosote, or a cloth, kept constantly wet with the solution, especially for the face.

The kreosote, when applied, should cause the parts to become white immediately. If this does not occur, it is not pure. Thus you will perceive that success depends upon having the best quality of oil. It is worthy of remark that the skin does not become in the least marked by the application, no matter how often it is applied.

I was first induced to make a trial of this remedy, by a remark made by Dupuytren in a small pamphlet which fell into my hands, in which he supposed it might be a good remedy in this disease.

The result of an extensive and exclusive use of this article in erysipelas, has induced me to place the most implicit confidence in it; and all I ask of the profession is a fair trial for it, confident that whoever once tries it, will abandon all other articles in its favor.—*Am. Jour. Med. Science.*.

